# Evaluation of qPCR reference genes in two genotypes of *Populus* for use in photoperiod and low-temperature studies

**DOI:** 10.1186/1756-0500-5-366

**Published:** 2012-07-23

**Authors:** Emily A Pettengill, Cécile Parmentier-Line, Gary D Coleman

**Affiliations:** 1 Department of Plant Science and Landscape Architecture, University of Maryland, College Park, Maryland, 20742-4452, USA

**Keywords:** RT-qPCR, Reference gene validation, *Populus trichocarpa*, *Populus tremula x Populus alba*

## Abstract

**Background:**

Quantitative PCR (qPCR) is a widely used technique for gene expression analysis. A common normalization method for accurate qPCR data analysis involves stable reference genes to determine relative gene expression. Despite extensive research in the forest tree species *Populus*, there is not a resource for reference genes that meet the Minimum Information for Publication of Quantitative Real-Time PCR Experiments (MIQE) standards for qPCR techniques and analysis. Since *Populus* is a woody perennial species, studies of seasonal changes in gene expression are important towards advancing knowledge of this important developmental and physiological trait. The objective of this study was to evaluate reference gene expression stability in various tissues and growth conditions in two important *Populus* genotypes (*P. trichocarpa* “Nisqually 1” and *P. tremula* x *P. alba* 717 1-B4) following MIQE guidelines.

**Results:**

We evaluated gene expression stability in shoot tips, young leaves, mature leaves and bark tissues from *P. trichocarpa* and *P. tremula. x P. alba* grown under long-day (LD), short-day (SD) or SD plus low-temperatures conditions. Gene expression data were analyzed for stable reference genes among *18S rRNA*, *ACT2*, *CDC2*, *CYC063*, *TIP4-like*, *UBQ7*, *PT1* and *ANT* using two software packages, geNorm^PLUS^ and BestKeeper. GeNorm^PLUS^ ranked *TIP4-like* and *PT1* among the most stable genes in most genotype/tissue combinations while BestKeeper ranked *CDC2* and *ACT2* among the most stable genes.

**Conclusions:**

This is the first comprehensive evaluation of reference genes in two important *Populus* genotypes and the only study in *Populus* that meets MIQE standards. Both analysis programs identified stable reference genes in both genotypes and all tissues grown under different photoperiods. This set of reference genes was found to be suitable for either genotype considered here and may potentially be suitable for other *Populus* species and genotypes. These results provide a valuable resource for the *Populus* research community.

## Background

Grown for timber, paper and bioenergy, the forest tree genus *Populus* is one of the most widely cultivated tree genera and has become a model for tree research [1]. Within this genus, two genotypes, *P. trichocarpa* and the hybrid *P. tremula x P. alba* are frequently used in molecular and genomic research. *P. trichocarpa* (Torr. And Gray) genotype ‘Nisqually-1’ has become a vital resource since completion of genome sequence [2] while *P. tremula x P. alba* clone INRA no. 717-1B4 is widely used for molecular biology research because of the ease and efficiency of *in vitro* shoot regeneration and genetic transformation methods [3]. These two genotypes have been extensively used to study seasonal nitrogen cycling and storage, SD associated growth cessation, leaf senescence, bud development and dormancy [4-11]. Identifying stable reference genes in various tissues in plants grown in both SD and LD conditions will help facilitate future research of seasonal traits in *Populus* using qPCR.

Results from qPCR assays and the conclusions based on qPCR data, have been an invaluable source for studying gene expression yet the broad application of qPCR methods requires standards that promote accuracy, reproducibility and transparency. There has been rapid adoption of a specific set of standards termed the Minimum Information for the Publication of Real-time Quantitative PCR Experiments (MIQE) [12-14]. The MIQE guidelines are a set of ideal practices for qPCR experiments that aim to reduce the publication of inaccurate data that could be interpreted to make incorrect or misleading scientific conclusions. The scope of the guidelines is extensive and includes stipulations for experimental design, sample acquisition, preparation and quality control, reverse transcription and qPCR reactions and data analysis. The guidelines also encompass rules related to nomenclature, particularly using the term quantification cycle (Cq) instead of threshold cycle (Ct) and the term reference genes as opposed to housekeeping genes [12]. Despite the wide acceptance of the need for experimental and publication standards, Gutierrez et al. [15] and Guenin et al. [16] note that plant biology research has been slow to adopt these standards and these guidelines are often ignored in publications.

An important component of the MIQE guidelines is the appropriate analysis of raw fluorescence data to normalize technical variation. A routine method incorporates data from stable reference genes to calculate relative gene expression. Stable reference genes are generally defined as genes with uniform transcript abundance across all samples that is above background fluorescence levels [17]. This is determined by statistical analyses that estimate gene expression stability for a set of candidate reference genes. Data for stable reference genes can then be included in normalization analyses [16]. QPCR validation is crucial for accurate data analysis and involves techniques that test if fluorescence data are a direct measure of gene expression in experimental samples [12]. This concept is illustrated by PCR amplification efficiencies (E), which are calculated by quantifying the increase of amplified product after each thermocycle in samples with a range of transcript abundance [12,18]. For example, aberrant product synthesis due to enzymatic inhibitors or secondary structures of the primers may not reflect the actual transcript quantity [18,19]. PCR efficiency values for each primer pair are included in calculations for stability and relative gene expression analyses [20,21].

Two reports that fail to conform to the publication standards outlined in the MIQE guidelines have been published evaluating reference genes for qPCR analysis in *Populus*[22,23]. The first report by Brunner et al. [22], omits the PCR efficiencies for each primer pair as well as the size of the amplification product. This work used ANOVA and linear regression techniques that have been supplanted by the availability of advanced statistical programs that rank reference gene stability [20,21]. In the second report by Xu et al. [23], all efficiencies are outside of the range of acceptable efficiencies (E = 1.9-2.1), indicative of possible unreliable product amplification that questions the validity of the findings [18,24]. Besides the technical aspects of these previous studies, both studies also used interspecific hybrids *(P. deltoides x P. nigra* or *P. trichocarpa x P. deltoides*) to conduct the analysis. Because of the lack of a detailed report of qPCR reference genes that conform to MIQE guidelines in poplar we conducted a MIQE compliant examination of reference genes in two poplar genotypes that are extensively used in genomic and transgenic studies, *P. trichocarpa* (Nisqually-1) and *P. tremula x P. alba* clone 717 1-B4.

In this study we report on the gene expression stability of 8 candidate reference genes (*18S rRNA*, *ACT2*, *CDC2*, *CYC063*, *TIP4-like*, *ANT*, *UBQ7*, and *PT1*) in 4 different tissues from plants grown under various photoperiodic conditions. Analyses were performed with the software packages geNorm^PLUS^ and BestKeeper. The results of this study provide a resource for *Populus* researchers and demonstrates the use of MIQE guidelines to the study of poplar gene expression.

## Results

### Candidate reference genes selection, PCR efficiency and expression profiles

To evaluate candidate reference genes for gene expression studies in *P. trichocarpa* and *P. tremula x P. alba*, qPCR assays were performed on triplicate biological samples from shoot tips, young leaves, mature leaves and bark at 5 time points under long day or short day photoperiods and short day photoperiods supplemented with low-temperatures. Reference genes were selected from existing literature on *Populus* (Table1).

**Table 1:** List and description of candidate reference genes for qPCR

**Symbol**	**Locus name Phytozome v2.2**	***At*ortholog accession no.**	**Gene name**	**Function**	**Reference**
*TIP4-like*	POPTR_0009s09620	NM_119592	TIP4-like	Putative cytoskeletal protein	[15]
*CYC063*	POPTR_0005s26170	AY652862^a^	Cyclophilin	Peptidylprolyl isomerase, protein folding	[25]
*PT1*	POPTR_0014s03160	NM_119492	Unknown protein	Unknown function, expressed in pollen tube cells	[15]
*CDC2*	POPTR_0004s14080	NM_114734	Cell division control protein 2	Cyclin-dependent kinase 2	[26]^b^
*ACT2*	POPTR_0001s31700	AB067722	Actin 2	Formation of filaments, component of cytoskeleton	[26]^b^
*18S rRNA*	Scaffold 17	AY652861^a^	18S ribosomal RNA	Constituent of ribosome	[25,27]
*ANT*	POPTR_0014s01260	AY117207	AINTEGUMENTA	Putative ovule development protein	[15]^b^
*UBQ7*	POPTR_0005s09940	NM_129118	Ubiquitin	Protein modification, ubuquitin-dependant protein catabolism	[22]

Gene symbol, *Populus* locus name (Phytozome), NCBI *Arabidopsis thaliana* ortholog accession number, gene name, function (annotation from Phytozome) and reference for each gene.

^a^ accession number for *Populus*.

^b^ primers were not redesigned.

**Figure 1:**
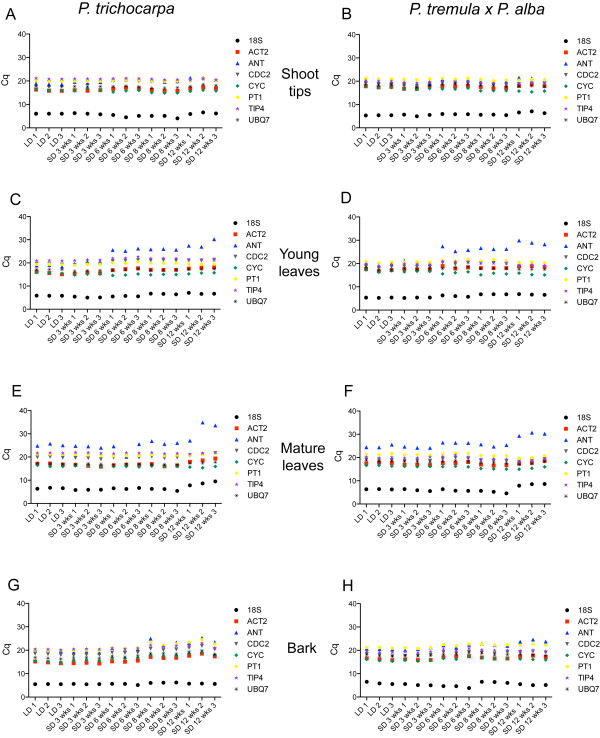
**Cq distributions for each candidate reference gene.** Expression data for reference genes where each graphed point represents the mean of the technical replicates. Each graph shows the quantification cycle (Cq) distribution for candidate reference genes in shoot tips/buds, young leaves, mature leaves and bark of both genotypes (*P. trichocarpa* and *P. tremula x P. alba*). LD, long day photoperiod; SD, short day photoperiod.

PCR efficiencies were calculated from the slopes of standard curves for all primer pairs and were found to be within the acceptable range of E = 1.9-2.1 for both *P. trichocarpa* and *P. tremula x P. alba* (Table2). Comparison of the same primer pairs between each genotype showed that the efficiencies were similar. The largest difference in PCR efficiencies between genotypes was 0.049 (or 4.9%) for *TIP4-like* and the smallest was 0.002 (or 0.2%) for *CYC063*. Expression levels of the candidate reference genes, presented in quantification cycle (Cq) values, showed that transcripts for all reference genes were detected in all samples for all tissues (Figure1). Cq values are the number of cycles when fluorescence crosses a threshold above background levels [12]. As shown in Figure1, the mean Cq values of all reference genes clustered together, around 20 cycles, except for *18S rRNA* where very low mean Cq values were observed around 5 cycles, indicating large transcript abundance. Furthermore, the Cq values for *ANT* tended to show greater variance than the other candidate genes, which is particularly evident in young and mature leaves of SD treated plants (Figure1, C, D, E, F). Shoot tips/buds and bark samples exhibited the least variation in mean Cq values of all genes amongst all the tissues.

**Table 2:** Characteristics of qPCR primers pairs for candidate reference genes

**Gene**	**Primers (5'-3')**	**Product size (bp)**	**Annealing temp (°C)**	***P. trichocarpa*PCR efficiency**	***P. tremula x P. alba*PCR efficiency**
*TIP4-like*	F: GCTGATAATGGGGTGTCG R: CAACTCTAAGCCAGAATCGC	88	57	1.969	2.018
*CYC063*	F: CCTGGCACTAATGGGTCTCAG R: CACAACTCTTCCGAACACCAC	87	52	1.98	1.978
*PT1*	F: GCGGAAAGAAAAACTGCAAG R: TGACAGCACAGCCCAATAAG	126	57	2.025	2.083
*CDC2*	F: ATTCCCCAAGTGGCCTTCTAAG R: TATTCATGCTCCAAAGCACTCC	137	57	2.04	2.035
*ACT2*	F: TTCTACAAGTGCTTTGATGGTGAGTTC R:CTATTCGATACATAGAAGATCAGAATGTTC	159	52	1.935	1.951
*18S rRNA*	F: GATTCTATGGGTGGTGGTGC R: CAGGCTGAGGTCTCGTTCG	87	60	1.951	1.965
*ANT*	F: TCTGTCTGTTATGCCCCTCA R: CCACCTAGGAAGTCCTCCAGT	119	55	2.062	2.033
*UBQ7*	F: GGAACGGGTTGAGGAGAAAGAAG R: GCAAGAACAAGATGAAGCACAGAGC	135	55	2.028	2.016

Primer sequences, PCR product sizes, annealing temperatures, PCR amplification efficiencies in *P. trichocarpa* and *P. tremula x P. alba* for each candidate reference gene.

### GeNorm^PLUS^ analyses

**Figure 2:**
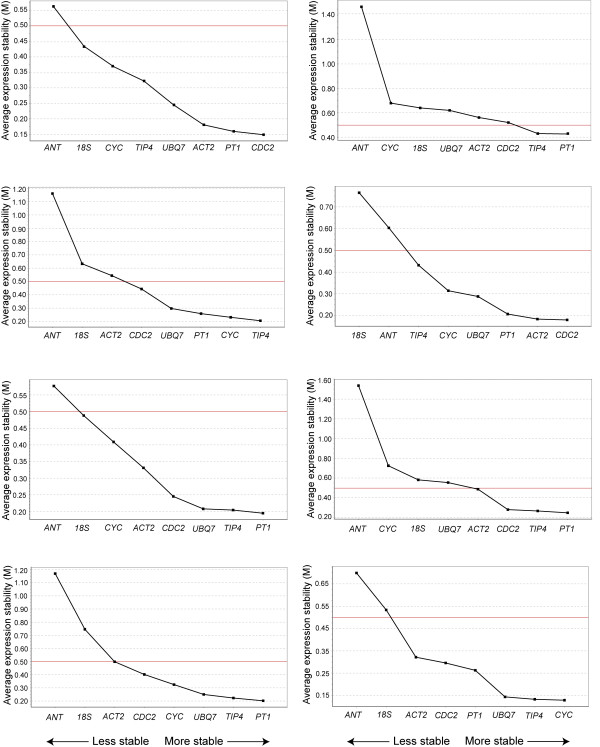
**Average expression stability values (M) and ranking of candidate reference genes determined by geNorm**^**PLUS**^**.** Candidate reference genes ordered from least stable (left) to most stable (right) in shoot tips/buds, young leaves, mature leaves and bark of both genotypes (*P. trichocarpa* and *P. tremula x P. alba*). The red line indicates the limit above which genes are considered non-stable (M = 0.5).

We determined the expression stability of the candidate reference genes using the geNorm^PLUS^ program within qbase^PLUS^ version 3. In these analyses we assumed that none of the selected genes were co-regulated since this is a prerequisite for geNorm^PLUS^ analysis. GeNorm^PLUS^ calculates the average gene expression stability (M) from the variation of the expression ratios of each pair of reference genes. This is based on the theory that two stable genes should share an identical expression ratio in all samples [21,28]. Lower M values indicate more stable gene expression with an upper threshold of M = 0.5, above which the reference genes are not considered stable. GeNorm^PLUS^ ranked the candidate reference genes according to their M values, from least stable to most stable (Figure2). *PT1* was ranked within the top three most stable genes for 7 out of the 8 genotype/tissue combinations and *TIP4-like* was ranked within the top three most stable genes for 5 out of the 8 genotype/tissue combinations. *ANT* and *18S rRNA* were ranked as the least stable genes in 6 out of the 8 genotype/tissue combinations. Ranking profiles differed for the same tissues between the two *Populus* genotypes. Genes were ranked at the same position in only 12 instances when comparing the two genotypes. Compared to other tissues, bark showed the greatest variation in stability ranking between the two genotypes of the reference genes. In contrast, young leaves showed the most similarities with 5 genes ranking at the same position for both genotypes: *PT1* and *TIP4-like* as the most stable and *18S rRNACYC063* and *ANT* as the least stable*.*

**Figure 3:**
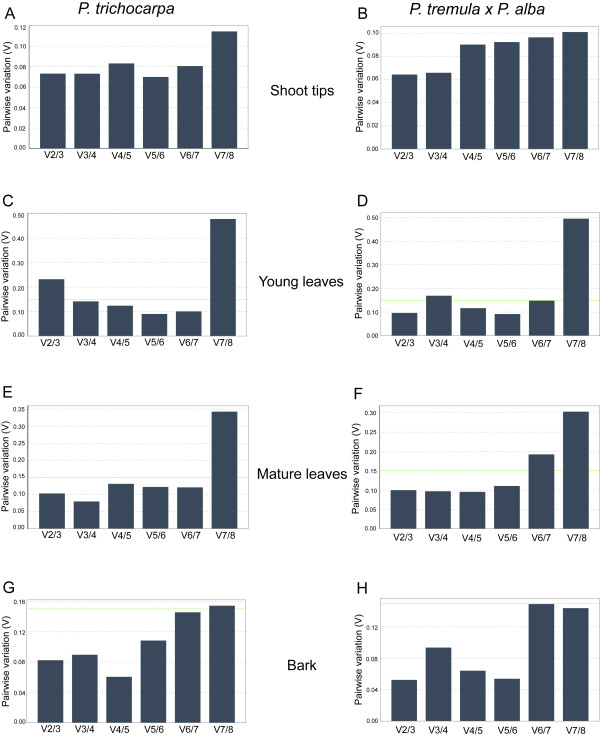
**Determination of optimal number of reference genes by geNorm**^**PLUS**^**.** Pairwise variation (V) analyses were performed to determine the optimum number of reference genes for normalization. V2/3 is the pairwise variation between the 2 most stable genes and the 3 most stable genes. V3/4 compares the 3 most stable genes with the 4 most stable genes, etc. The green line indicates the variation cut-off (V = 0.15) below which additional genes are not required for adequate normalization.

GeNorm^PLUS^ also determines the minimum number of reference genes to include in normalization analysis by calculating the average pairwise variation (V) of normalization factors which is determined by the two most stable genes and the addition of the next most stable gene until all genes have been added [28]. It has a cut-off value of 0.15, below which the addition of another reference gene has no significant effect and is not required. For samples of young leaves from *P. trichocarpa*, pairwise variation analysis showed that normalization should be performed with 3 reference genes since the V2/3 value was higher than 0.15 (Figure3). For all other tissues, the two most stable reference genes were sufficient to give a V value below 0.15.

### BestKeeper analyses

BestKeeper determines stable expression by first calculating descriptive statistics for each reference gene using the mean Cq data of the technical replicates for each sample. Then, using pairwise correlation analysis, the program compares each reference gene to the *BestKeeper Index* (*BKI*) and calculates a Pearson’s correlation coefficient (*r*) and *p*-value [20]. Higher correlation coefficients suggest more stable expression. Table3 shows the ranking of reference genes with corresponding (*r*) and *p-*values as determined by BestKeeper. *CDC2* was one of the 3 most stable genes in 7 of the 8 genotype/tissue combinations (*r* ≥ 0.718, *p*-value = 0.001). *ACT2* was ranked as one of the 3 most stable genes in 5 of the 8 genotype/tissue combinations (*r* ≥0.862, *p*-value = 0.001). Conversely, BestKeeper ranked *TIP4-like* as the least or second least stable gene in 6 out of 8 genotype/tissue combinations with the lowest correlation values of *r* = 0.057 (*p*-value = 0.837) in mature leaves of *P. tremula x P. alba*. Despite a low ranking in the bark of *P. trichocarpaTIP4-like* expression had a high correlation coefficient and significant *p*-value (*r* = 0.957, *p*-value = 0.001) when compared to the *BKI*. Rankings between the different tissues of the two genotypes were very distinct. Pfaffl et al. [20] recommend that if the standard deviation of the mean Cq values for replicates for a reference gene is greater than 1 then the data is considered inconsistent and calculations should be performed again without these genes. We observed standard deviations greater than 1 in all genotype/tissue combinations except shoot tips (Additional file 1: Table S1). These reference genes were removed and the data was reanalyzed. Removing these genes did not change the overall rankings for the remaining genes but resulted in increased correlation coefficients (*r*) for most of the remaining reference genes (Additional file 1: Table S1).

**Table 3:** Pairwise correlation analyses using BestKeeper

***Populus trichocarpa***											
**Shoot tips/buds**	**Coeff. of corr. (*r*)**	***p*-value**	**Young leaves**	**Coeff. of corr. (*r*)**	***p*-value**	**Mature leaves**	**Coeff. of corr. (*r*)**	***p*-value**	**Bark**	**Coeff. of corr. (*r*)**	***p*-value**
*UBQ7*	0.892	0.001	*CDC2*	0.970	0.001	*18S rRNA*	0.964	0.001	*CDC2*	0.993	0.001
*18S rRNA*	0.810	0.001	*ANT*	0.963	0.001	*ACT2*	0.962	0.001	*PT1*	0.993	0.001
*CDC2*	0.718	0.003	*ACT2*	0.961	0.001	*CDC2*	0.943	0.001	*ACT2*	0.983	0.001
*PT1*	0.660	0.007	*18S rRNA*	0.799	0.001	*ANT*	0.917	0.001	*UBQ7*	0.980	0.001
*ACT2*	0.655	0.008	*PT1*	0.610	0.016	*UBQ7*	0.845	0.001	*ANT*	0.970	0.001
*CYC063*	0.639	0.010	*UBQ7*	0.532	0.041	*PT1*	0.663	0.007	*CYC063*	0.969	0.001
*ANT*	0.360	0.188	*TIP4-like*	0.517	0.048	*TIP4-like*	0.088	0.754	*TIP4-like*	0.957	0.001
*TIP4-like*	0.223	0.427	*CYC063*	−0.008	0.977	*CYC063*	−0.477	0.072	*18S rRNA*	0.650	0.009
***Populus tremula x Populus alba***											
**Shoot tips/buds**	**Coeff. of corr. (*r*)**	***p*-value**	**Young leaves**	**Coeff. of corr. (*r*)**	***p*-value**	**Mature leaves**	**Coeff. of corr. (*r*)**	***p*-value**	**Bark**	**Coeff. of corr. (*r*)**	***p*-value**
*CDC2*	0.905	0.001	*CDC2*	0.977	0.001	*ACT2*	0.894	0.001	*PT1*	0.812	0.001
*ACT2*	0.862	0.001	*ANT*	0.944	0.001	*18S rRNA*	0.816	0.001	*CDC2*	0.793	0.001
*ANT*	0.788	0.001	*18S rRNA*	0.886	0.001	*ANT*	0.653	0.008	*ANT*	0.738	0.002
*18S rRNA*	0.659	0.008	*ACT2*	0.881	0.001	*CDC2*	0.569	0.027	*ACT2*	0.650	0.009
*UBQ7*	0.651	0.009	*PT1*	0.562	0.029	*CYC063*	0.420	0.119	*CYC063*	0.647	0.009
*PT1*	0.562	0.029	*TIP4-like*	0.416	0.123	*UBQ7*	0.323	0.240	*UBQ7*	0.644	0.009
*TIP4-like*	0.457	0.087	*UBQ7*	0.358	0.191	*TIP4-like*	0.057	0.837	*TIP4-like*	0.633	0.011
*CYC063*	0.129	0.646	*CYC063*	−0.717	0.003	*PT1*	0.050	0.860	*18S rRNA*	0.389	0.152

Each candidate reference gene was compared to the *BestKeeper Index* to calculate a Pearson’s coefficient of correlation (*r*) and a *p*-value. Larger correlation coefficients indicate greater correlation with the index and is evidence of higher gene expression stability.

### GeNorm^PLUS^ versus BestKeeper

In comparing candidate reference gene stability rankings produced by geNorm^PLUS^ and BestKeeper, we found that these two programs ranked the reference genes differently (Table4). For instance, BestKeeper frequently assigned *ANT* a middle ranking and even ranked it as the second most stable gene in young leaves of both genotypes. On the other hand, geNorm^PLUS^ consistently ranked *ANT* as the least or second least stable gene in all tissues. The rankings of *18S rRNA* by geNorm^PLUS^ and BestKeeper also showed differences. *18S rRNA* ranked as the least or second least stable gene when analyzed by geNorm^PLUS^ while BestKeeper assigned *18S rRNA* a high or middle ranking except for bark tissues where it was ranked as one of the least stable genes. There were only 4 occurrences in *P. trichocarpa* where the two programs gave the same ranking for a gene: *CYC063* in shoot tips and *CDC2*, *UBQ7* and *18S rRNA* in bark. In all tissues of *P. tremula x P. alba* and in mature leaves of *P. trichocarpa*, the genes recommended by geNorm^PLUS^ for normalization calculations were ranked amongst the 4 least stable genes by BestKeeper.

**Table 4:** **Comparison of stability rankings between geNorm**^**PLUS**^** and BestKeeper**

***Populus trichocarpa***							
**Shoot tips/buds**		**Young leaves**		**Mature leaves**		**Bark**	
geNorm^PLUS^	BestKeeper	geNorm^PLUS^	BestKeeper	geNorm^PLUS^	BestKeeper	geNorm^PLUS^	BestKeeper
CDC2*	*UBQ7*	PT1*	*CDC2*	TIP4-like*	*18S rRNA*	CDC2*	*CDC2*
PT1*	*18S rRNA*	TIP4-like*	*ANT*	CYC063*	*ACT2*	ACT2*	*PT1*
*ACT2*	*CDC2*	CDC2*	*ACT2*	*PT1*	*CDC2*	*PT1*	*ACT2*
*UBQ7*	*PT1*	*ACT2*	*18S rRNA*	*UBQ7*	*ANT*	*UBQ7*	*UBQ7*
*TIP4-like*	*ACT2*	*UBQ7*	*PT1*	*CDC2*	*UBQ7*	*CYC063*	*ANT*
*CYC063*	*CYC063*	*18S rRNA*	*UBQ7*	*ACT2*	*PT1*	*TIP4-like*	*CYC063*
*18S rRNA*	*ANT*	*CYC063*	*TIP4-like*	*18S rRNA*	*TIP4-like*	*ANT*	*TIP4-like*
*ANT*	*TIP4-like*	*ANT*	*CYC063*	*ANT*	*CYC063*	*18S rRNA*	*18S rRNA*
***Populus tremula x Populus alba***							
		**Young leaves**		**Mature leaves**		**Bark**	
geNorm^PLUS^	BestKeeper	geNorm^PLUS^	BestKeeper	geNorm^PLUS^	BestKeeper	geNorm^PLUS^	BestKeeper
PT1*	*CDC2*	PT1*	*CDC2*	PT1*	*ACT2*	CYC063*	*PT1*
TIP4-like*	*ACT2*	TIP4-like*	*ANT*	TIP4-like*	*18S rRNA*	TIP4-like*	*CDC2*
*UBQ7*	*ANT*	*UBQ7*	*18S rRNA*	*UBQ7*	*ANT*	*UBQ7*	*ANT*
*CDC2*	*18S rRNA*	*CDC2*	*ACT2*	*CYC063*	*CDC2*	*PT1*	*ACT2*
*ACT2*	*UBQ7*	*ACT2*	*PT1*	*CDC2*	*CYC063*	*CDC2*	*CYC063*
*CYC063*	*PT1*	*18S rRNA*	*TIP4-like*	*ACT2*	*UBQ7*	*ACT2*	*UBQ7*
*18S rRNA*	*TIP4-like*	*CYC063*	*UBQ7*	*18S rRNA*	*TIP4-like*	*18S rRNA*	*TIP4-like*
*ANT*	*CYC063*	*ANT*	*CYC063*	*ANT*	*PT1*	*ANT*	*18S rRNA*

Candidate reference genes ordered from most stable (top) to least stable (bottom) for each tissue by geNorm^PLUS^ and BestKeeper in *P. trichocarpa* and *P. tremula x P. alba*. Reference genes indentified by geNorm^PLUS^ for inclusion in normalization calculations are indicated by * symbol.

## Discussion

Recent reports have questioned the validity of selecting reference genes for qPCR analysis of gene expression based on results from other species or different experimental regimes [15,16,29-31]. In this report we undertook a stability analysis of 8 reference genes expressed in various tissues of two genotypes of *Populus* grown in LD and SD conditions. The stability of the reference genes was then determined using two different programs: geNorm^PLUS^ and BestKeeper. In addition to these two programs, NormFinder is another program that measures reference gene expression stability [32]. Together, these are the three widely cited programs used for stability analysis. GeNorm^PLUS^ has been cited over 4,000 times, followed by NormFinder with over 650 citations and BestKeeper with over 500 citations (determined by Google Scholar search). In contrast to geNorm^PLUS^ and BestKeeper, NormFinder requires defining two or more groups of samples composed of at least eight samples per group for accurate analysis [32]. Since our experimental design did not meet these requirements NormFinder was not included in this study.

Irrespective of the analysis program used to determine reference gene stability, the most stable reference genes vary among tissues of both genotypes. Besides variation in gene expression stability between tissues within a genotype, it was also found that reference gene stability also varies between genotypes within a given tissue. For example, in shoot tips/buds *CDC2* was ranked by geNorm^PLUS^ as the most stable reference gene in *P. trichocarpa* but ranked as the fourth most stable reference gene in *P. tremula x P. alba* (Table4). BestKeeper ranked *UBQ*7 as the most stable reference gene in shoot tips/buds in *P. trichocarpa* and as the fifth most stable gene in *P. tremula x P. alba*. This difference of ranking in the same tissues of the two genotypes occurred regardless of the program used. Although, geNorm^PLUS^ rankings between genotypes of the least stable reference genes were more consistent than rankings of the most stable genes. Previous studies on coffee and petunia [33,34] also concluded that reference genes were different in different tissues for a single genotype and also for the same tissue between different genotypes. This variation in reference gene stability underscores the importance of empirically testing all samples in an experiment to validate reference gene stability.

This report is a rigorous evaluation of reference gene stability in *Populus* and a valuable resource when compared to previous reports in *Populus*[22,23]. Beside our adherence to the MIQE guidelines, there are additional distinctions between this report and previous reports. Brunner et al. [22] determined reference gene stability using ANOVA and linear regression analyses while we used currently available methods capable of more refined statistics. An additional difference between the current study and Brunner et al. [22] is that primers used in their study were designed from a limited number of ESTs, whereas we designed primers using sequences from the *Populus* genome, which is a more complete resource. Xu et al. [23] used the same programs we used to evaluate stable reference genes in bark and root tissues during adventitious root formation. However, the reported amplification efficiencies were outside the range suggested by the MIQE guidelines making it difficult to determine the accuracy of their stability rankings. Finally, in this report we performed reference gene evaluations using the two important *Populus* genotypes, *P. trichocarpa* “Nisqually 1” and *P. tremula* x *P. alba* 717 1-B4.

Consistent with prior reports, our results found that stability rankings were not consistent amongst geNorm^PLUS^ and BestKeeper programs [23,35,36]. These discrepancies are a consequence of the different statistical methods that the programs are based. BestKeeper performs pairwise correlation analysis using Cq values compared to an index value while geNorm^PLUS^ calculates the ratio of variation between pairs of reference genes. *ANT* is a good example of the differences between stability rankings. While *ANT* is not generally considered to be a reference gene, it was included in this study as a gene with documented variable expression in cambium [15]. The mean Cq distributions of *ANT* clearly confirm expression in all genotype/tissue combinations we studied making *ANT* a suitable candidate reference gene to test. The mean Cq distributions show that *ANT* expression is variable (Figure1). Consistent with the report of variable *ANT* expression, geNorm^PLUS^ ranked *ANT* as the overall least stable gene in all genotype/tissue combinations. Yet BestKeeper assigned, in most cases, a high rank to *ANT.* Although geNorm^PLUS^ ranked *ANT* as one of the least stable reference genes in both genotypes and range of tissues, there may be unique conditions in which *ANT* could be used as a reference gene. For example, the Cq distributions in bark in *P. trichocarpa* indicate that *ANT* appears stable in samples up to 6 weeks of SD exposure (Figure1, G). This could account for the high correlation coefficients of *ANT* (r = 0.970, *p* = 0.001) in this tissue type as calculated by BestKeeper (Table3). Additionally, geNorm^PLUS^ generally ranked the expression of *18S rRNA* as unstable in all genotype/tissue combinations while BestKeeper tended to rank this gene unstable in bark and more stable in the other tissues. The graphs of Cq distributions show that Cq values for *18S rRNA* do not appear to be as stable compared to the other reference genes (Figure1) and the Cq distributions more closely agree with the assigned rankings by geNorm^PLUS^ than by BestKeeper. This calls attention to the importance of reviewing the Cq distributions in conjunction with the ranking profiles by expression stability programs for confirmation of stability. Regardless of its stability*,* inclusion of *18S rRNA* as a reference gene for qPCR assays requires cDNA synthesized with random primers instead of oligodT primers. It is common to synthesize cDNA with oligodT primers to limit sample complexity when investigating differential expression by qPCR. Therefore, omitting *18S rRNA* as a reference gene would allow a researcher to maintain a low sample complexity when synthesizing cDNA. For those reasons we do not recommend *18S rRNA*.

The purpose of this study was not to provide specific reference gene recommendations but to offer a set of rigorously tested reference genes that are potentially suitable as reference genes for expression analyses in *Populus*. Testing the PCR efficiencies of primer pairs in both genotypes revealed that PCR efficiencies were similar although not identical yet within the acceptable range. It is probable that these primers may also be suitable for use in other *Populus* species provided that adequate PCR efficiencies are validated [37].

Researchers should carefully choose a gene stability analysis program that fits their experimental needs. Each program has limitations and specific requirements for analyses. For example, NormFinder requires at least 2 groups of 8 or more samples for accurate analyses [32]. This is significant because it can be difficult to define logical groups that comprise an adequate number of samples within a group. There are reports in which samples are grouped in multiple ways, which affected the calculations and rankings [38,39]. Results from BestKeeper can be difficult to interpret, as illustrated in this paper. High correlation coefficients and significant *p*-values can be calculated even for unstable reference genes. When considering geNorm^PLUS^, researchers should take into account that the program currently does not perform analyses for a reference gene if the Cq data were collected from more than one plate, which may be impractical for large studies. Therefore, the choice of analysis program must be appropriate for the experimental design.

The importance of using multiple reference genes for normalization analyses has long been established and including multiple reference genes for normalization is a component of MIQE guidelines [12,28]. One of the unique features of geNorm^PLUS^ is the ability to calculate the minimum number of reference genes to include in normalization analyses. In this study, analysis with geNorm^PLUS^ indicates that the 2 most stable reference genes were adequate for normalization analyses except for one case where 3 reference genes were recommended. This offers an advantage in accurate normalization calculations compared to analysis with NormFinder or BestKeeper. If using these programs, including 3 or more stable reference genes is suggested as a “universally applicable method” [17]. In this study, geNorm^PLUS^ is the program that best fits our experimental needs. It differentiates between biological and technical replicates and calculates the best number of reference genes needed for normalization. More practically, it is the most user-friendly program with clear indications of the most stable reference genes as well as integrated alerts that inform users of data errors or omissions.

## Conclusions

In this study it was possible to identify stable reference genes that can be employed to investigate changes in differential gene expression in *Populus* under controlled environments including LD, SD and SD with low temperatures. Rigorous testing of candidate reference genes can be time and energy intensive but it is crucial to obtaining valuable scientific conclusions. Here we provide a set of established reference genes for which we tested the normalization potential in a study of their expression stability in two poplar genotypes. We also conclude that geNorm^PLUS^ is the most useful program to determine the stability of reference genes. It calculates stability based on rigorous statistical methods, and integrates calculations to determine the appropriate number of reference genes for normalization and it is user-friendly. This report emphasizes the importance of the MIQE recommendations and promotes the continued adoption of the recommendations by researchers studying *Populus*.

## Methods

### Plant material

*P. trichocarpa* (Nisqually-1) plants were grown from cuttings prepared from greenhouse grown plants. *P. tremula x P. alba* clone (717 1-B4) plants were propagated using *in vitro* shoot cultures and rooted plantlets. Plants of both genotypes were grown in 2.2 L pots containing a commercial potting mix (Sunshine LC1) and fertilized with approximately 5 g of the slow release fertilizer (Nutricote, 18-3-3; Florikan, Sarasota, FL, USA). All photoperiod studies were conducted in controlled environment chambers (Conviron Inc., Winnipeg, Manitoba, Canada) at 18°C with a PAR at 50 cm above the surface of pots, ranging from 310–470 μmol m^-2^ s^-1^.

To study the effect of changing photoperiods, plants were grown for 8 weeks in long-days (LD;16 h light/8 h dark) followed by short-days (SD; 8 h light/16 h dark) for an additional 12 weeks. During the last 4 weeks in SD, the temperature was lowered to 10°C day/4°C night. Various tissues were collected at 5 time points: 8 weeks LD and after 3, 6, 8 and 12 weeks SD. The tissues included apical shoot tips/buds, bark (between leaf plastochron index 8 and 9 [LPI 8–9]), young leaves (LPI 3) and mature leaves (LPI 9). Samples were immediately frozen in liquid N_2_ and stored at −80°C until used for RNA extraction. Triplicate biological samples were composed of the pooled tissues from 4 individuals (total of 12 plants).

### Design and validation of qPCR primers

Primers were designed using MacVector version 11 (MacVector Inc., Cary, NC, USA) based on the following criteria: 18–25 nucleotides in length, GC content of 40-60%, product length ~60-150 bp, and designed to amplify products within 500 bp of the 3’ end [19,24]. Primers were tested for optimum annealing temperature using a temperature gradient and for specificity with a melt curve. PCR amplification efficiencies for all primer pairs were calculated by the iQ5 software (Bio-Rad, Hercules, CA, USA) from a five-point calibration curve of ten-fold serial dilutions. Melt curves were performed for every run to confirm amplification of a single product.

### RNA extraction, cDNA synthesis and qPCR detection

Total RNA was extracted using the RNeasy plant mini kit with the automated QIAcube (Qiagen, Valencia, CA, USA). Samples were ground in liquid N_2_ with a mortar and pestle. RLT buffer containing 1% beta-mercaptoethanol and 1% polyvinylpyrrolidone was added to 50 mL tubes containing ground tissue and vortexed thoroughly. Following suspension in the modified RLT buffer, 0.4 volumes 5 M potassium acetate, pH 6.5 was added to the buffer, mixed by inverting and incubated for 15 min on ice. Samples were centrifuged for 15 min at 15,000 *g* at 4°C. Supernatant was then loaded into the QIAcube and RNA extraction was performed with an on-column DNAse I (Qiagen, Valencia, CA, USA) digestion. RNA quality and quantity was assessed with microfluidics using the Experion™ automated electrophoresis system and RNA StdSens chips (Bio-Rad, Hercules, CA, USA). cDNA synthesis reactions were performed with 1 μg of total RNA and oligodT primers according to manufacturer’s instructions (RevertAid, Fermentas Inc., Glen Burnie, MD, USA). Separate reactions were performed for *18S rRNA* using random primers instead of oligodT primers. The cDNA from triplicate first strand cDNA reactions was pooled and served as the template for triplicate technical qPCR reactions with the Maxima SYBR green qPCR master mix (Fermentas Inc., Glen Burnie, MD, USA) and detected with the iQ5 Real-Time PCR Detection System (Bio-Rad, Hercules, CA, USA). Cycling conditions consisted of 10 min at 95°C followed by 40 cycles of 15 sec at 95°C and 1 min at the optimum annealing temperature (Table2).

### Statistical analyses

Data from the iQ5 Real-Time Detection System (Bio-Rad, Hercules, CA, USA) were analysed with geNorm^PLUS^ in qbase^PLUS^ version 3 (http://www.qbaseplus.com) and BestKeeper version 1 (http://gene-quantification.com/bestkeeper.html) [20].

## Competing interests

The authors declare that they have no competing interests.

## Authors’ contributions

EAP designed primers, designed experiments, collected and analyzed data and wrote the manuscript. CPL extracted and analyzed RNA, designed experiments, collected and analyzed data and edited the manuscript. GDC oversaw the project and edited the manuscript. All authors read and approved the final manuscript.

## Supplementary Material

Additional file 1 **BestKeeper input and output data.** Cq distributions, descriptive statistics, pairwise correlation analysis tables, correlation coefficient values for first and second analyses. Click here for file

## References

[B1] Taylor G Populus: arabidopsis for forestry. Do we need a model tree? Ann Bot 2002 90 6 681 10.1093/aob/mcf255 12451023 PMC4240366

[B2] Tuskan GA Difazio S Jansson S Bohlmann J Grigoriev I Hellsten U Putnam N Ralph S Rombauts S Salamov A The genome of black cottonwood, Populus trichocarpa (Torr. and Gray) Science 2006 313 5793 1596 1604 10.1126/science.1128691 16973872

[B3] Leple J Brasileiro A Michel M Delmotte F Jouanin L Transgenic poplars-expression of chimeric genes using 4 different constructs Plant Cell Reports 1992 11 3 137 141 10.1007/BF00232166 24213546

[B4] Davis J Egelkrout E Coleman G Chen T Haissig B Riemenschneider D Gordon M A family of wound-induced genes in Populus shares common features with genes encoding vegetative storage proteins Plant MolBiol 1993 23 1 135 143 10.1007/BF00021426 8106009

[B5] Zhu B Coleman G Phytochrome-mediated photoperiod perception, shoot growth, glutamine, calcium, and protein phosphorylation influence the activity of the poplar bark storage protein gene promoter (bspA) Plant Physiol 2001 126 1 342 351 10.1104/pp.126.1.342 11351097 PMC102308

[B6] Zhu B Coleman G The poplar bark storage protein gene (Bspa) promoter is responsive to photoperiod and nitrogen in transgenic poplar and active in floral tissues, immature seeds and germinating seeds of transgenic tobacco Plant MolBiol 2001 46 4 383 394 10.1023/a:1010600504740 11485196

[B7] Bhalerao R Keskitalo J Sterky F Erlandsson R Bjorkbacka H Birve S Karlsson J Gardestrom P Gustafsson P Lundeberg J Gene expression in autumn leaves Plant Physiol 2003 131 2 430 442 10.1104/pp.012732 12586868 PMC166820

[B8] Andersson A Keskitalo J Sjodin A Bhalerao R Sterky F Wissel K Tandre K Aspeborg H Moyle R Ohmiya Y A transcriptional timetable of autumn senescence Genome Biol 2004 5 4 R24 10.1186/gb-2004-5-4-r24 15059257 PMC395783

[B9] Druart N Johansson A Baba K Schrader J Sjödin A Bhalerao RR Resman L Trygg J Moritz T Bhalerao RP Environmental and hormonal regulation of the activity-dormancy cycle in the cambial meristem involves stage-specific modulation of transcriptional and metabolic networks The Plant Journal 2007 50 4 557 573 10.1111/j.1365-313X.2007.03077.x 17419838

[B10] Ruttink T Arend M Morreel K Storme V Rombauts S Fromm J Bhalerao RP Boerjan W Rohde A A molecular timetable for apical bud formation and dormancy induction in poplar The Plant Cell 2007 19 8 2370 2390 10.1105/tpc.107.052811 17693531 PMC2002631

[B11] Resman L Howe G Jonsen D Englund M Druart N Schrader J Antti H Skinner J Sjodin A Chen T Components acting downstream of short day perception regulate differential cessation of cambial activity and associated responses in early and late clones of hybrid poplar Plant Physiol 2010 154 3 1294 1303 10.1104/pp.110.163907 20847139 PMC2971607

[B12] Bustin S Benes V Garson J Hellemans J Huggett J Kubista M Mueller R Nolan T Pfaffl M Shipley G The MIQE guidelines: minimum information for publication of quantitative real-time PCR experiments Clin Chem 2009 55 4 611 10.1373/clinchem.2008.112797 19246619

[B13] Bustin S Why the need for qPCR publication guidelines? The case for MIQE Methods 2010 50 4 217 226 10.1016/j.ymeth.2009.12.006 20025972

[B14] Bustin SA Benes V Garson JA Hellemans J Huggett J Kubista M Mueller R Nolan T Pfaffl MW Shipley GL Primer sequence disclosure: a clarification of the MIQE guidelines Clin Chem 2011 57 919 921 10.1373/clinchem.2011.162958 21421813

[B15] Gutierrez L Mauriat M Guenin S Pelloux J Lefebvre J-F Louvet R Rusterucci C Moritz T Guerineau F Bellini C The lack of a systematic validation of reference genes: a serious pitfall undervalued in reverse transcription-polymerase chain reaction (RT-PCR) analysis in plants Plant Biotech Journal 2008 6 6 609 618 10.1111/j.1467-7652.2008.00346.x 18433420

[B16] Guenin S Mauriat M Pelloux J Van Wuytswinkel O Bellini C Gutierrez L Normalization of qRT-PCR data: the necessity of adopting a systematic, experimental conditions-specific, validation of references Journal Exp Botany 2009 60 2 487 493 10.1093/jxb/ern305 19264760

[B17] Derveaux S Vandesompele J Hellemans J How to do successful gene expression analysis using real-time PCR Methods 2010 50 4 227 230 10.1016/j.ymeth.2009.11.001 19969088

[B18] Taylor S Wakem M Dijkman G Alsarraj M Nguyen M A practical approach to RT-qPCR–Publishing data that conform to the MIQE guidelines Methods 2010 50 4 S1 S5 10.1016/j.ymeth.2010.01.005 20215014

[B19] Udvardi MK Czechowski T Scheible W-R Eleven golden rules of quantitative RT-PCR Plant Cell 2008 20 7 1736 1737 10.1105/tpc.108.061143 18664613 PMC2518243

[B20] Pfaffl M Tichopad A Prgomet C Neuvians T Determination of stable housekeeping genes, differentially regulated target genes and sample integrity: BestKeeper–Excel-based tool using pair-wise correlations Biotech letters 2004 26 6 509 515 10.1023/b:bile.0000019559.84305.47 15127793

[B21] Hellemans J Mortier G De Paepe A Speleman F Vandesompele J qBase relative quantification framework and software for management and automated analysis of real-time quantitative PCR data Genome Biol 2007 8 R19 10.1186/gb-2007-8-2-r19 17291332 PMC1852402

[B22] Brunner A Yakovlev I Strauss S Validating internal controls for quantitative plant gene expression studies BMC Plant Biology 2004 4 14 10.1186/1471-2229-4-14 15317655 PMC515301

[B23] Xu M Zhang B Su X Zhang S Huang M Reference gene selection for quantitative real-time polymerase chain reaction in Populus Analytical Biochem 2011 408 2 337 339 10.1016/j.ab.2010.08.044 20816740

[B24] Real-time PCR applications guide http://www.xpcrx.com/data/working/library/1_DNA/REFERENCE%20-%20Real-Time_PCR_Applications_Guide_(0612_RevB).pdf

[B25] Brentner L Mukherji S Merchie K Yoon J Schnoor J Aken B Expression of glutathione S-transferases in poplar trees (Populus trichocarpa) exposed to 2, 4, 6-trinitrotoluene (TNT) Chemosphere 2008 73 5 657 662 10.1016/j.chemosphere.2008.07.059 18774158

[B26] Nicole M-C Hamel L-P Morency M-J Beaudoin N Ellis BE Seguin A MAP-ping genomic organization and organ-specific expression profiles of poplar MAP kinases and MAP kinase kinases BMC Genomics 2006 7 223 10.1186/1471-2164-7-223 16945144 PMC1574314

[B27] Lu S Zhou Y Li L Chiang VL Distinct roles of cinnamate 4-hydroxylase genes in Populus Plant and Cell Physiology 2006 47 7 905 914 10.1093/pcp/pcj063 16720648

[B28] Vandesompele J De Preter K Pattyn F Poppe B Van Roy N De Paepe A Speleman F Accurate normalization of real-time quantitative RT-PCR data by geometric averaging of multiple internal control genes Genome Biol 2002 3 7 RESEARCH0034 10.1186/gb-2002-3-7-research0034 PMC126239 12184808

[B29] Chen L Zhong H-Kuang J-Li J-Lu W-Chen J-Validation of reference genes for RT-qPCR studies of gene expression in banana fruit under different experimental conditions Planta 2011 234 2 377 390 10.1007/s00425-011-1410-3 21505864

[B30] Fernandez P Di Rienzo JA Moschen S Dosio GAA Aguirrezábal LAN Hopp HE Paniego N Heinz RA Comparison of predictive methods and biological validation for qPCR reference genes in sunflower leaf senescence transcript analysis Plant Cell Reports 2011 30 1 63 74 10.1007/s00299-010-0944-3 21076836

[B31] Vashisth T Johnson LK Malladi A An efficient RNA isolation procedure and identification of reference genes for normalization of gene expression in blueberry Plant Cell Reports 2011 30 12 2167 2176 10.1007/s00299-011-1121-z 21761237

[B32] Andersen C Jensen J Orntoft T Normalization of real-time quantitative reverse transcription-PCR data: A model-based variance estimation approach to identify genes suited for normalization, applied to bladder and colon cancer data sets Cancer Res 2004 64 15 5245 5250 10.1158/0008-5472.CAN-04-0496 15289330

[B33] Barsalobres-Cavallari CF Severino FE Maluf MP Maia IG Identification of suitable internal control genes for expression studies in Coffea arabica under different experimental conditions BMC MolBiol 2009 10 1 10.1186/1471-2199-10-1 PMC2629470 19126214

[B34] Mallona I Lischewski S Weiss J Hause B Egea-Cortines M Validation of reference genes for quantitative real-time PCR during leaf and flower development in Petunia hybrida BMC Plant Biology 2010 10 4 10.1186/1471-2229-10-4 20056000 PMC2827423

[B35] Tong Z Gao Z Wang F Zhou J Zhang Z Selection of reliable reference genes for gene expression studies in peach using real-time PCR BMC MolBiol 2009 10 71 10.1186/1471-2199-10-71 PMC3224724 19619301

[B36] Lin YL Lai ZX Reference gene selection for qPCR analysis during somatic embryogenesis in longan tree Plant Science 2010 178 359 365 10.1016/j.plantsci.2010.02.005

[B37] Boyle B Dallaire N MacKay J Evaluation of the impact of single nucleotide polymorphisms and primer mismatches on quantitative PCR BMC Biotechnol 2009 9 1 75 10.1186/1472-6750-9-75 19715565 PMC2741440

[B38] Exposito-Rodriguez M Borges AA Borges-Perez A Perez JA Selection of internal control genes for quantitative real-time RT-PCR studies during tomato development process BMC Plant Biology 2008 8 131 10.1186/1471-2229-8-131 19102748 PMC2629474

[B39] Huis R Hawkins S Neutelings G Selection of reference genes for quantitative gene expression normalization in flax (Linum usitatissimum L.) BMC Plant Biology 2010 10 71 10.1186/1471-2229-10-71 20403198 PMC3095345

